# Spatial and Seasonal Variations in the Abundance of Nitrogen-Transforming Genes and the Microbial Community Structure in Freshwater Lakes with Different Trophic Statuses

**DOI:** 10.3390/ijerph16132298

**Published:** 2019-06-28

**Authors:** Yu Wan, Xiaohong Ruan, Jie Wang, Xiaojun Shi

**Affiliations:** 1 College of Resources and Environment, Southwest University, Chongqing 400716, China; 2 School of Earth Science and Engineering, Nanjing University, Nanjing 210023, China; 3 Academy of Agricultural Sciences, Southwest University, Chongqing 400716, China

**Keywords:** nitrogen-transforming genes, function microorganism, trophic status, sediment, Lake Taihu

## Abstract

Identifying nitrogen-transforming genes and the microbial community in the lacustrine sedimentary environment is critical for revealing nitrogen cycle processes in eutrophic lakes. In this study, we examined the diversity and abundance of ammonia-oxidizing bacteria (AOB), ammonia-oxidizing archaea (AOA), denitrifying bacteria (DNB), and anammox bacteria (AAOB) in different trophic status regions of Lake Taihu using the *amoA*, *Arch-amoA*, *nirS*, and *hzo* genes as functional markers. Quantitative Polymerase Chain Reaction (qPCR) results indicated that the abundance of the *nirS* gene was the highest, while the *amoA* gene had the lowest abundance in all regions. Except for the primary inflow area of Lake Taihu, *Arch-amoA* gene abundance was higher than the *hzo* gene in three lake bays, and the abundance of the *nirS* gene increased with decreasing trophic status. The opposite pattern was observed for the *amoA*, *Arch-amoA*, and *hzo* genes. Phylogenetic analyses showed that the predominant AOB and AOA were *Nitrosomonas* and *Nitrosopumilus maritimus*, respectively, and the proportion of *Nitrosomonas* in the eutrophic region (87.9%) was higher than that in the mesotrophic region (71.1%). *Brocadia* and *Anammoxoglobus* were the two predominant AAOB in Lake Taihu. Five novel unknown phylotypes of AAOB were observed, and Cluster AAOB-B was only observed in the inflow area with a proportion of 32%. In the DNB community, *Flavobacterium* occurred at a higher proportion (22.6–38.2%) in all regions, the proportion of *Arthrobacter* in the mesotrophic region (3.6%) was significantly lower than that in the eutrophic region (15.6%), and the proportions of Cluster DNB-E in the inflow area (24.5%) was significantly higher than that in the lake bay (7.3%). The canonical correspondence analysis demonstrated that the substrate concentration in sedimentary environments, such as NO_x_^-^-N in the sediment, NH_4_^+^-N in the pore water, and the total organic matter, were the key factors that determined the nitrogen-transforming microbial community. However, the temperature was also a predominant factor affecting the AOA and AAOB communities.

## 1. Introduction

Nitrogen is a limiting nutritional factor in the primary productivity of lakes and is one of the primary factors contributing to the eutrophication of lakes [1]. As the primary driving force of the nitrogen cycle, nitrogen-transforming bacteria affect the occurrence of various forms of nitrogen and their characteristics [2]. Therefore, research on the nitrogen-transforming bacterial community and gene abundance in a lake aquatic environment is not only significant to elucidate nitrogen cycle and accumulation mechanisms in the eutrophic lakes but also provides a theoretical basis for preventing and controlling nitrogen pollution of eutrophic lakes.

Nitrite reductase (*nir)* catalyzes NO_2_^−^ reduction to NO, and is the limiting step in denitrification [3]. The *nir* gene has two types *(nirS* and *nirK)*, with *nirS* being more widely distributed than *nirK* in lake sediments [4]. Therefore, using the *nirS* gene to research the denitrifying bacteria (DNB) was more representative than the *nirK* gene. Because the ammonia-oxidizing bacteria (AOB) *amoA* gene is strongly conserved and could be detected in all AOB, the *amoA* gene is one of the most representative molecular markers for research on the AOB [5]. Venter et al. (2004) recently found that ammonia-oxidizing archaea (AOA) also have *amoA* genes that function in a similar fashion to the AOB and played an even larger role than the AOB [6]. Because the anaerobic ammonia oxidation bacteria (AAOB) 16S rRNA gene may underestimate its diversity (nonspecific amplification cannot cover all phylogenetic branches) [7], the *hzo* gene has been considered to be the most appropriate marker for an AAOB phylogenetic analysis in recent years [8]. As a result, those functional genes—*nirS*, *amoA*, and *hzo*—represent the main driving force of the nitrogen cycle and nitrogen transformation.

Considerable recent research shows that various environmental factors could lead to changes in the abundance of microbial nitrogen-transforming genes and the community structure [9,10,11]. A study by Sun et al. (2014) found that the abundance of the AOB and AOA *amoA* gene in eight major freshwater lakes of Jiangsu province showed significant spatial differences under the influence of the sediment TOC and C/N [12]. Lisa et al. (2015) researched *hzo* and *amoA* gene abundance in the sediments of the Cape Fear River Estuary and observed that *hzo* gene abundance was higher nearer to the estuary, and salt-resistant bacterial species appeared [13]. Henry et al (2006) investigated the abundance of denitrification functional genes in wetland sediments and cropland soils in different countries and noted that under the influence of environmental factors, the abundance of denitrification functional genes was significantly different in the different research areas [14]. Because of the river input pollution load and the lake flow field, a series of nitrogen concentration and nutrient levels gradient was present in Lake Taihu from the north (Meiliang Bay) to the southeast (East Lake Taihu). At present, although numerous studies have investigated nitrogen-transforming genes in these sediments, they have generally focused only on a single nitrogen-transforming bacterium. By amplifying the AAOB 16S rRNA gene, Wu et al. (2012) found that the AAOB existed in the sediments of Lake Taihu, but the main operational taxonomic unit (OTU) was an unknown and unclassified AAOB [15]. Dai et al. (2013) and Wu et al. (2010) noted that a significant difference was present in the diversity of the AOB and the AOA and the abundance of *amoA* genes in the sediments in Lake Taihu, the diversity of the AOA was lower than the AOB, but the AOA *amoA* gene abundance was higher than the AOB [16,17]. Guo et al. (2014) compared the *nirS*-type DNB community structure between Meiliang Bay and five other lakes with different trophic statuses and observed that trophic status significantly affected the distribution and diversity of the DNB [18]. The authors further observed that the diversity of the DNB in the severely eutrophic Meiliang Bay was significantly lower than in the other lakes, and the DNB community structure in Meiliang Bay was significantly different from other lakes [18]. These investigations helped to establish a broad understanding of the distribution patterns of nitrogen-transforming genes and the microbial community in this lake environment. However, the relationship between the nitrogen-transforming bacterial community structure, gene abundance and nitrogen occurrence characteristics in lake regions with different trophic statuses needs to be studied thoroughly.

Therefore, the aim of this study was to dissect the nitrogen-transforming bacterial community structure and gene abundance (1) to determine the relationship between the sediment bacterial taxa and the trophic status of the lake water and sedimentary environmental factors, (2) and to provide powerful evidence for further elucidation of the nitrogen cycle and accumulation mechanisms driven by bacteria in aquatic ecosystems.

## 2. Materials and Methods

### 2.1. Sampling Site and Procedure

**Figure 1:**
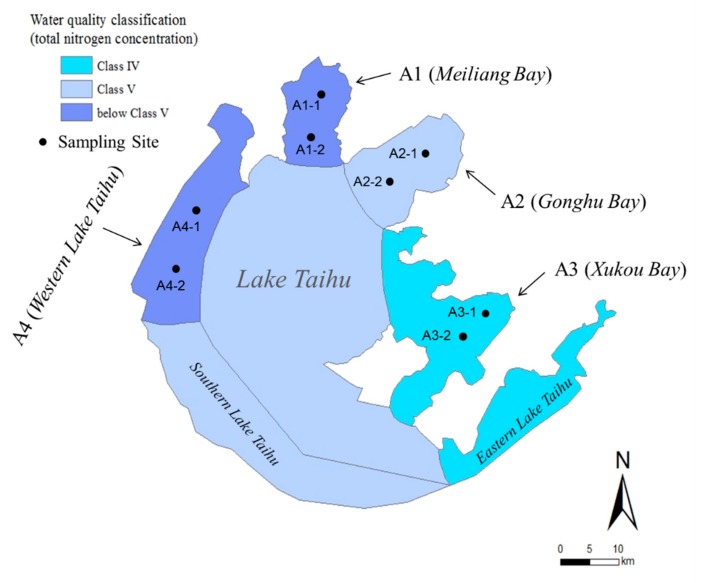
The sampling sites in the different trophic statuses of the lake. Water quality classification of the lake regions in Lake Taihu referred to the Environmental Quality Standard for Surface Water (GB3838-2002). Classification standard value: 1 < TN ≤ 1.5, 1 < NH_4_^+^-N ≤ 1.5 were categorized as Class IV; 1.5 < TN ≤ 2, 1.5 < NH_4_^+^-N ≤ 2 were categorized as Class V; TN > 2, NH_4_^+^-N > 2 were categorized as below Class V. The data are sourced from The Health Status Report of Lake Taihu 2014 [19].

The study was conducted in the north, east, and west sides of Lake Taihu (Figure 1), and the total nitrogen decreases from Meiliang Bay (region A1, north) and Western Lake Taihu (region A4, west) to Gonghu Bay (region A2, northeast) and, finally, to Xukou Bay (region A3, east). Each lake region had 2 sampling sites. Areas A1 and A4 are highly nutrient-enriched and have frequent algal blooming incidents. Area A4 is the main the inflow area of Lake Taihu, which has received large amounts of municipal wastewater. In contrast, the low nutrient waterbody in A3 is characterized by submerged vegetation and diverse communities of fishes and invertebrates, and is a drinking water source for the local community. The water at A2 was similar to that at A1 and A4 until approximately 15 years ago, although its quality has since improved.

Sample collection was carried out in March (spring), June (summer), September (autumn), and December (winter) 2016. The temperatures in March, June, September, and December 2016 were 16, 33, 25, and 8 °C, respectively, during sampling. Overlying water was collected below 1 m of the lake surface using an organic glass hydrophore. Three water samples were aseptically collected at each sampling site into sterile 1-L sampling bottles. After water collection, sediment samples were then collected at the sampling point as the water. For sediments, samples at a maximum depth of 5 cm, and were collected using a 1/16-m^2^ Petersen grab sampler. Triplicate samples from three separate grabs were homogenized to generate a composite sample (approximately 6 kg) for each sampling site, and put into sterile plastic bags. All samples were immediately stored in an icebox at 4 °C and transported back to the laboratory within 3 h. Once in the laboratory, an aliquot of the sediment sample (1 kg) was aseptically stored at −80 °C until DNA extraction was performed. The remaining portion (5 kg) of sediments was further processed for as described in the analytical methods of soil agrochemistry [20] for the analysis of the sediments and pore water physicochemical properties.

### 2.2. Physicochemical Analysis

Fourteen physicochemical parameters of the overlying water, pore water and freeze-dried sediments were analyzed (Table S1). Details of the measurement procedures for each parameter in sediment, pore water, and overlying water can be found in Lu (1999) and Wei (2002), respectively [20,21]. The temperature (T), chlorophyll a (Chla), and pH of the overlying water were measured in situ using a YSI-6600-V2 Multi-Parameter Water Quality Sonde (YSI Incorporated, Yellow Springs, OH, USA), and the transparency was determined by a standard Secchi disc (SD) (diameter 20 centimeters) with black and white quadrants.

### 2.3. PCR Amplification, Clone Libraries Construction, and Phylogenetic Analysis 

The total genomic DNA of each sediment sample was extracted using a Powersoil DNA extraction kit (Mo Bio Laboratories) according to the manufacturer’s instructions. PCR amplification of *hzo* genes in the AAOB bacteria, *amoA* genes in the AOB, *Arch-amoA* genes in the AOA, and *nirS* genes in the DNB were determined according to previously established protocols [5,22,23,24], and the PCR primers used for the PCR amplification are shown in Table S2. To understand the community composition of the AAOB, AOA, AOB and the DNB in the research samples, clone libraries of each target gene in the sample were constructed. Triplicate PCR reaction products for each site were pooled, gel purified using an E.Z.N.A.^®^ Cycle-Pure Kit (Omega Bio-tek Inc., Norcross, GA, USA), and cloned using the pMD18 T-vector (Takara Bio Inc., Kusatsu, Shiga, Japan). 

Screened clones were sequenced using an ABI Prism genetic analyzer (Applied Biosystems, Foster City, CA, USA) in combination with a BIG Dye Terminator kit (Applied Biosystems, Canada). The functional gene sequences were edited, and the vector sequences were clipped using the DNAstar software package (DNASTAR, Madison, WI, USA). We checked for possible chimeras using the CHECK CHIMERA program from the Ribosomal Database Project (http://rdp.cme.msu.edu/). The gene sequences were analyzed initially using the BLASTn tool (http://www.ncbi.nlm.nih.gov/BLAST/) to facilitate the selection of the closest reference sequences. Sequences displaying more than 97% identity with one another were grouped into one OTU using the Mothur software 1.23.0 (http://sourceforge.net/projects/seqclean/ and http://www.mothur.org/wiki/MainPage). Neighbor-joining phylogenetic trees were created with one representative sequence of each OTU, and the reference sequences were retrieved from GenBank using the Molecular Evolutionary Genetics Analysis (MEGA) software 5.03 (Center for Evolutionary Medicine and Informatics, The Biodesign Institute, Tempe, AZ, USA). The relative confidence of the tree topologies was evaluated by performing 1000 bootstrap replicates.

### 2.4. Real-Time Quantitative PCR

Quantitative PCR (qPCR) was performed using an ABI 7500 FAST (Applied Biosystems, Foster City, CA, USA) to determine the *amoA* (AOB), *Arch-amoA* (AOA), *hzo* (AAOB), and *nirS* (DNB) genes copy number. Primer sets of *amoA*1f/*amoA*2r, *Arch-amoA*F/*Arch-amoA*R, *hzo*F1/*hzo*R1, and *nirS*3F/*nirS*5R were used for the determination of the bacterial and archaeal *amoA*, *hzo*, and *nirS* genes, respectively, with the SYBR green-based reactions being performed in triplicate for each sample as described previously [25]. The qPCR standard was generated using plasmid DNA from representative clones containing the microbial nitrogen-transforming genes. A dilution series of the standard template across 7 orders of magnitude (10^1^ to 10^7^) for nitrogen-transforming genes was used in each assay. A No Template Control was always run with water as the template instead of sediment DNA extract. The amplification efficiency and coefficient (*r*^2^) for the *amoA*, *Arch-amoA*, *hzo*, and *nirS* genes were, respectively, 91.2 and 0.992, 92.6 and 0.995, 90.5 and 0.993, 93.8 and 0.998%.

### 2.5. Statistical Analysis

The Trophic Status Indices (*TSI*s) for all of the sampling sites were calculated using the measured Chla, W-TP, W-TN, COD, and SD by the following expression:

(1) $$TSI\left(\sum \right)=\overset{m}{\underset{i=1}{\sum }}w_{j}\;TSI\left(j\right)$$

where $TSI\left(\sum^{\text{​}}\right)$ is the completed *TSI*, $w_{j}$ is the relative weight of the *TSI* of the *jth* parameter, and TSI(j) is the *TSI* of the *jth* parameter. The mean value of all of the samples from each region was used to represent the local trophic status which, based on the value of $TSI\left(\sum^{\text{​}}\right)$, can be classified as oligotrophic (0 < *TSI* ≤ 30), mesotrophic (30 < *TSI* ≤ 50), lightly eutrophic (50< *TSI* ≤ 60), medium eutrophic (60 < *TSI* ≤ 70), and hypereutrophic (70 < *TSI* ≤ 100) on a scale of 0 to 100.

The indices of diversity were calculated for each constructed gene library using the Mothur software (version 1.23.0, USA). The coverage of each clone library (C) was calculated according to Mullins et al. (1995) [26].

(2) $$C=100\%\left[1-\left(\frac{n}{N}\right)\right]$$

where *n* is the number of unique OTUs, and *N* is the total number of clones in a library.

We used detrended correspondence analysis (DCA) implemented in CANOCO 4.5 (Biometris-Plant Research International, Wageningen, The Netherlands) for gradient length diagnostics. Gradient lengths of the first DCA axes of DNB, AOB, AOA, and AAOB OTUs were 3.71, 3.42, 3.22, and 3.35, respectively. The correlations between nitrogen-transforming bacterial community structure and eight environmental factors were determined by canonical correspondence analysis (CCA) using CANOCO 4.5. *p* < 0.05 was considered significant correlation, and *p* < 0.1 was considered weak correlation. Among the eight environmental factors, six factors (*TSI*, S-TN, S-NH_4_, S-NO_x_, P-NH_4_, and P-NO_3_) are associated to nitrogen, and S-TOM is related to the carbon source required for microbial growth, while the temperature reflects the seasonal variation. 

## 3. Results

### 3.1. Physicochemical Properties of the Lake Regions

The average water temperature of the four studied lake regions in March, June, September, and December were 11.2, 27.1, 20.3, and 3.5 °C, respectively. Measured *TSI* in the study area decreased in the direction of A1, A2, and A3 with average values changing from 61.0, 56.3, to 45.3, respectively (Figure S1, Table S3), corresponding to the state of medium eutrophication, light eutrophication, and mesotrophication. A4 was in the state of light eutrophication in winter and medium eutrophication in the other three seasons. For pore water, while the average value of NH_4_^+^-N decreased in the same direction as *TSI* (Figure S1-a), the concentration of NO_3_^−^-N in the research area did not show significant variation (Figure S1-b). Similar to the trend of the NH_4_^+^-N in the pore water, the content of NH_4_^+^-N (Figure S1-c) and NO_x_^−^-N (Figure S1-d) in the sediments decreased with decreasing trophic status.

### 3.2. The Abundance of Microbial Nitrogen-Transforming Genes

**Figure 2:**
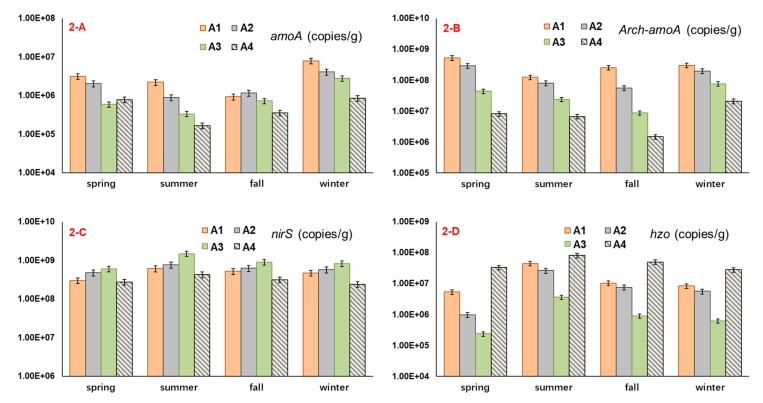
The average value of nitrogen-transforming gene abundances in each lake region during four seasons. **2-A**: *amoA* gene, **2-B**: *Arch*-*amoA* gene, **2-C**: *nirS* gene, **2-D**: *hzo* gene.

A quantitative PCR analysis of *nirS*, *amoA*, *Arch*-*amoA*, and *hzo* gene abundance is shown in Figure 2. The abundance of each nitrogen-transforming gene at two sampling sites in each lake region was slightly different. The abundance of *nirS* in regions A1, A2, A3, and A4, respectively, ranged from 2.55 × 10^8^–7.65 × 10^8^, 4.10 × 10^8^–8.92 × 10^8^, 4.74 × 10^8^–1.76 × 10^9^, and 1.49 × 10^8^–5.14 × 10^8^ copies/g (Table S4). The abundance of *amoA* in regions A1, A2, A3, and A4, respectively, ranged from 5.96 × 10^5^–9.40 × 10^6^, 7.27 × 10^5^–4.91 × 10^6^, 2.69 × 10^5^–3.74 × 10^6^, and 1.31 × 10^5^–1.17 × 10^6^ copies/g (Table S5). The abundance of *Arch*-*amoA* in regions A1, A2, A3, and A4, respectively, ranged from 9.42 × 10^7^–6.32 × 10^8^, 4.43 × 10^7^–3.42 × 10^8^, 6.62 × 10^6^–1.02 × 10^8^, and 9.52 × 10^5^–2.84 × 10^7^ copies/g (Table S6). The abundance of *hzo* in regions A1, A2, A3, and A4, respectively, ranged from 4.54 × 10^6^–5.61 × 10^7^, 8.48 × 10^5^–3.06 × 10^7^, 1.86 × 10^5^–4.33 × 10^6^, and 1.74 × 10^7^–9.83 × 10^7^ copies/g (Table S7). 

A comparison of the abundance of the nitrogen-transforming functional genes in the research area showed that the *nirS* gene abundance was the highest, and the *amoA* gene abundance was the lowest. The abundance of the nitrogen-transforming functional genes varied in the different lake sediments. Except for region A4, the *nirS* gene abundance decreases with an increase in the lake trophic status at the other three lake regions, and the abundance of *amoA*, *Arch*-*amoA* and *hzo* genes changed in the opposite direction (Figure 2). The seasonal changes in the abundance of nitrogen-transforming functional genes showed that the abundance of the *nirS* and *hzo* genes in summer and autumn was greater than that in the spring and the winter, and the *amoA* and *Arch*-*amoA* gene abundance showed an opposite trend (Figure 2).

### 3.3. Phylogenetic Analyses of the Nitrogen-Transforming Microbes

The *nirS*, *amoA*, *Arch*-*amoA*, and *hzo* gene segments were amplified successfully in the summer and the winter. A total of 218 *nirS*, 185 *amoA*, 201 *Arch*-*amoA*, and 164 *hzo* clones were sequenced from the 32 constructed clone libraries (Table 1). Within each individual clone library, 12 to 19 DNB, 4 to 11 AOB, 3 to 5 AOA, and 5 to 10 AAOB OTUs occurred, as defined by a <3% divergence in the nucleotides. The diversity of the DNB *nirS* was higher than the AOB *amoA*, the AOA *Arch*-*amoA*, and the AAOB *hzo* genes in each lake region based on the values of the Shannon-Wiener. The DNB *nirS* gene had a higher diversity in the winter than in the summer, whereas the seasonal variation of the diversity of the AOA *amoA* gene was not significant (Table 1).

**Figure 3:**
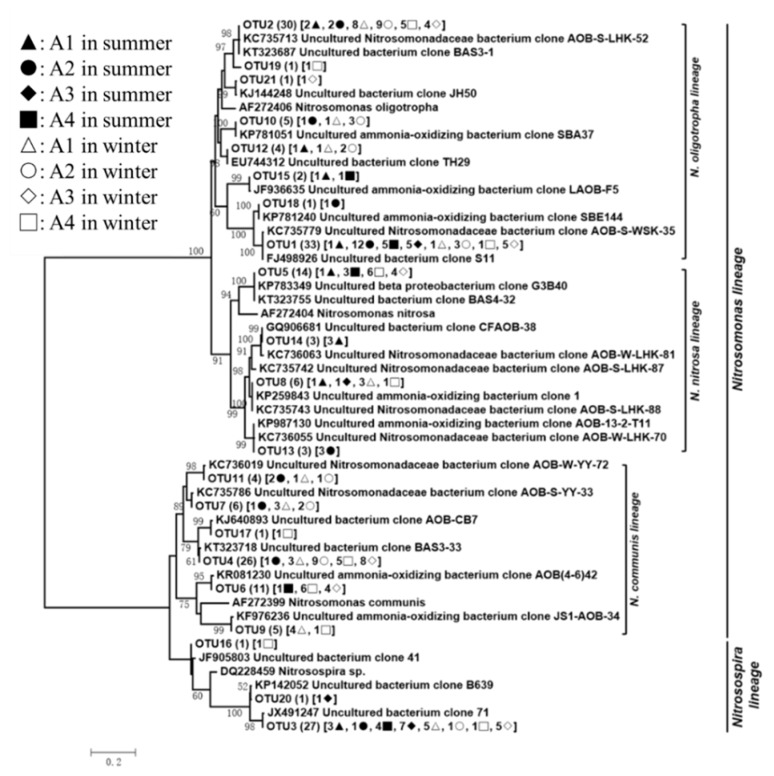
Neighbor-joining phylogenetic tree based on the nucleotide sequences of the *amoA* gene. The numbers at the nodes represent the credible values that were calculated from 1000-times bootstrap tests. Bootstrap values greater than 50% were shown. The scale bar indicated 0.2 substitutions per nucleotide position. GenBank accession numbers were shown for sequences from other studies. Numbers in parentheses following each OTU indicated the number of sequences recovered from each sampling site.

**Figure 4:**
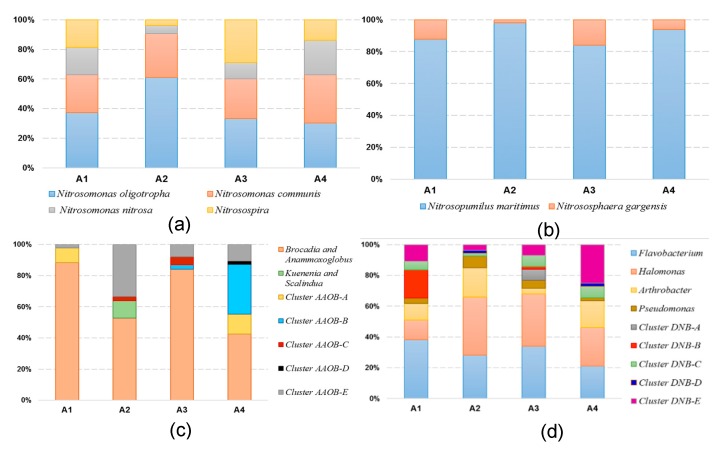
The relative abundance of (**a**) AOB *amoA* gene, (**b**) AOA *Arch*-*amoA* gene, (**c**) AAOB *hzo* gene, and (**d**) DNB *nirS* gene clone libraries at four regions.

The 185 *amoA* gene sequences had 71.2–100.0% sequence similarity with each other and had high degrees of identity (96.7–100.0%) to the most closely matching GenBank sequences. The *amoA* sequences were grouped into 21 unique OTUs, but most of them were not closely matched with sequences from any known AOB isolates. A phylogenetic analysis indicated that the *amoA* sequences grouped with known sequences from the *Nitrosomonas* AOB (18 OTUs and 156 clones) and *Nitrosospira* (DG228459; three OTUs and 29 clones) genera (Figure 3). All of the *Nitrosomonas*-related sequences grouped with sequences in the lineage of *Nitrosomonas oligotropha* (AF272406; eight OTUs and 77 clones), *Nitrosomonas nitrosa* (AF272404; four OTUs and 26 clones), and *Nitrosomonas communis* (AF272399; six OTUs and 53 clones). Four of the 21 *amoA* OTUs were common, including OTU1 (33 clones, *Nitrosomonas oligotropha*) and OTU3 (27 clones, *Nitrosospira*), which occurred at all of the eight sampling points, OTU2 (30 clones, *Nitrosomonas oligotropha*) and OTU4 (26 clones, *Nitrosomonas communis*), which respectively occurred at six and five sampling points. Although these OTUs represented only 19.1% of all of the AOB OTUs identified, they accounted for 63.0% of all of the clones recovered. As shown in Figure 4a, *Nitrosomonas* was the predominant AOB in Lake Taihu and was more significantly predominant in the eutrophic lake regions. The relative abundance of the *Nitrosospira* significantly increased in the mesotrophic lake region. *Nitrosomonas oligotropha* and *Nitrosomonas communis* were predominant AOBs in A1, A2, and A4, which respectively encompassed 63, 91, and 63% of the AOB group, while *Nitrosomonas oligotropha* and *Nitrosospira* were predominant in A3, which encompassed 62% of the AOB group.

**Figure 5:**
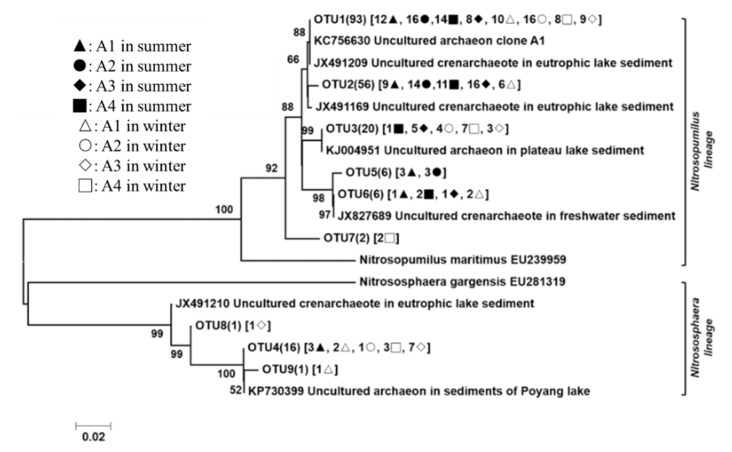
Neighbor-joining phylogenetic tree based on the nucleotide sequences of the *Arch*-*amoA* gene. The numbers at the nodes represented the credible values that were calculated from 1000-times bootstrap tests. Bootstrap values greater than 50% were shown. The scale bar indicated 0.02 substitutions per nucleotide position. GenBank accession numbers were shown for sequences from other studies. Numbers in parentheses following each OTU indicated the number of sequences recovered from each sampling site.

The 201 *Arch-amoA* gene sequences had 81.2–99.8% sequence similarity with each other and had high degrees of identity (97.1–100.0%) to the most closely matching GenBank sequences. Phylogenetic analysis indicated that the *Arch-amoA* sequences grouped with known sequences from the AOA in *Nitrosopumilus maritimus* (EU239959; six OTUs, 183 clones) and *Nitrososphaera gargensis* (EU281319; three OTUs, 18 clones) (Figure 5). Three of the nine *Arch-amoA* OTUs were common, including OTU1 (93 clones), which occurred at all eight sampling points, OTU2 (30 clones) and OTU3 (26 clones) at five sampling points. The three omnipresent OTUs were affiliated with the *Nitrosopumilus maritimus* lineage. Although these OTUs represented only 33.3% of all of the AOA OTUs identified, they accounted for 84.1% of all of the clones recovered. As shown in Figure 4b, *Nitrosopumilus maritimus* was the predominant AOA in Lake Taihu, and respectively encompassed 88, 98, 84, and 94% of the AOA group in A1, A2, A3, and A4, and *Nitrososphaera gargensis* was almost only observed in sampling sites of winter.

**Figure 6:**
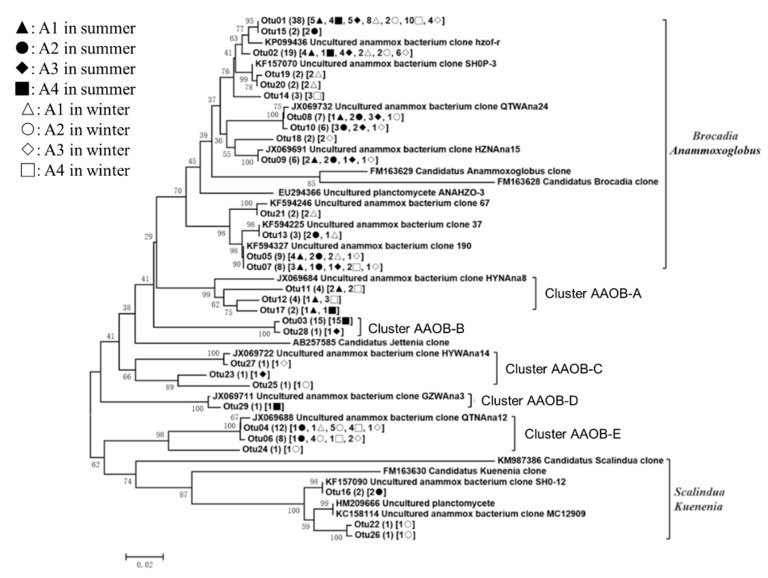
Neighbor-joining phylogenetic tree based on the nucleotide sequences of the *hzo* gene. The numbers at the nodes represented the credible values that were calculated from 1000-times bootstrap tests. Bootstrap values greater than 20% were shown. The scale bar indicated 0.02 substitutions per nucleotide position. GenBank accession numbers were shown for sequences from other studies. Numbers in parentheses following each OTU indicated the number of sequences recovered from each sampling site.

The 164 *hzo* gene sequences had 84.5–99.2% sequence similarity with one another and had a high degree of identity (95.3–99.4%) to the most closely matching GenBank sequences. A phylogenetic analysis indicated that the *hzo* sequences grouped with known sequences from the AAOB in *Kuenenia* and *Scalindua* (KM987386 and KF163630; 14 OTUs, 110 clones), *Brocadia* and *Anammoxoglobus* (FM163629 and KF594246; three OTUs, four clones), and cluster AAOB A-E (Figure 6). Three of the 29 *hzo* OTUs were common, including OTU1 (38 clones), which occurred at seven sampling plots, OTU2 (19 clones) and OTU4 (12 clones), which respectively occurred at six and five sampling plots. OTU1 and OTU2 were affiliated with the *Brocadia* and *Anammoxoglobus* lineage. As shown in Fig. 4c, *Brocadia* and *Anammoxoglobus* were the two predominant AAOBs in Lake Taihu, which respectively encompassed 88, 53, 84, and 43% of the AAOB group in A1, A2, A3, and A4, and showed more significant abundance in A1 and A3. A phylogenetic analysis of the AAOB suggests that the clusters of AAOB A-E may represent novel phylotypes of the anammox bacteria. Cluster AAOB-E and cluster AAOB-B were the other abundant anammox bacteria in A2 and A4, respectively. Moreover, a small number of *Kuenenia* and *Scalindua* was only found in A2, and cluster AAOB-B was only found in the inflow area at a proportion up to 32%.

**Figure 7:**
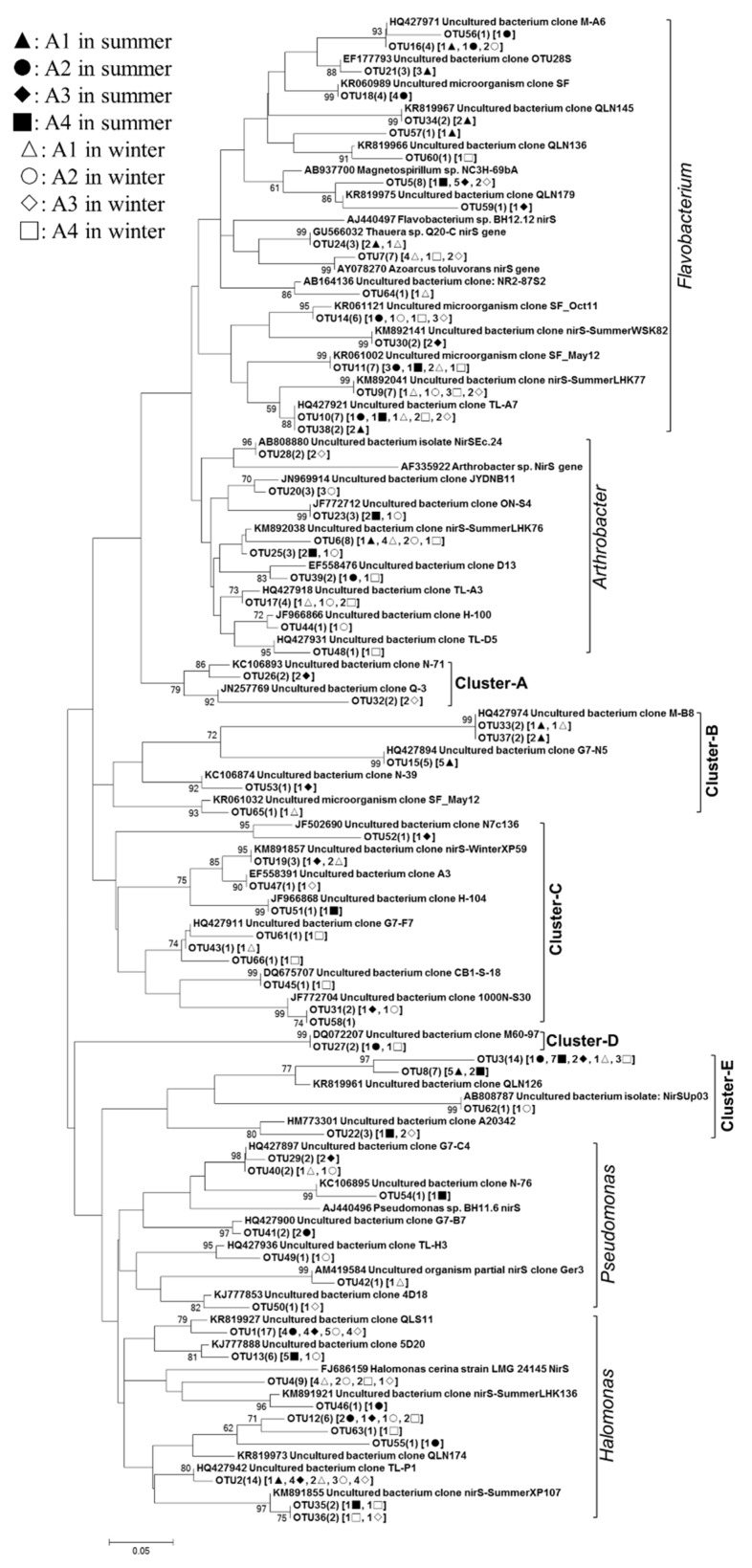
Neighbor-joining phylogenetic tree based on the nucleotide sequences of the *nirS* gene. The numbers at the nodes represented the credible values that were calculated from 1000-times bootstrap tests. Bootstrap values greater than 50% were shown. The scale bar indicated 0.05 substitutions per nucleotide position. GenBank accession numbers were shown for sequences from other studies. Numbers in parentheses following each OTU indicated the number of sequences recovered from each sampling site.

The 218 *nirS* gene sequences had 88.1–99.5% sequence similarity to each other and a high degree of identity (96.5–99.1%) to the most closely matching GenBank sequences. A phylogenetic analysis indicated that the *nirS* sequences grouped with known sequences from the DNB in *Flavobacterium* (AJ440497; 18 OTUs, 67 clones), *Arthrobacter* (AF335922; 9 OTUs, 27 clones), *Pseudomonas* (AJ440496; seven OTUs, 10 clones), *Halomonas* (FJ686159; 10 OTUs, 59 clones) and Cluster DNB A-E (Figure 7). Two of the 66 *nirS* OTUs were common, including OTU1 (14 clones) and OTU3 (14 clones), which all occurred at five sampling points. OTU2 and OTU3 were, respectively, affiliated with the *Halomonas* and Cluster DNB-E lineages. As shown in Figure 4d, *Flavobacterium* and Cluster DNB-B predominated in 56% of the DNB group in A1, *Flavobacterium* and *Halomonas* predominated in 66% and 68% of the DNB group in A2 and A3, respectively, and *Halomonas* and Cluster DNB-E predominated in 50% of the DNB group in A4. The proportion of *Arthrobacter* in the mesotrophic region (3.6%) was significantly lower than in the eutrophic region (15.6%), and the proportion of Cluster DNB-E in the inflow area (24.5%) was significantly higher than in the lake bay (7.3%).

### 3.4. Relationships between Microbial Community Composition and Environmental Variables

The environmental factors in the lake regions with different trophic statuses led to differences in the nitrogen-transforming microbial community structure. We chose temperature, *TSI*, S-TN, S-NH_4_, S-NO_x_, S-TOM, P-NH_4_, and P-NO_3_ to determine the primary factors that affected the nitrogen-transforming microbial community structure in the lake regions with different trophic statuses. 

**Figure 8:**
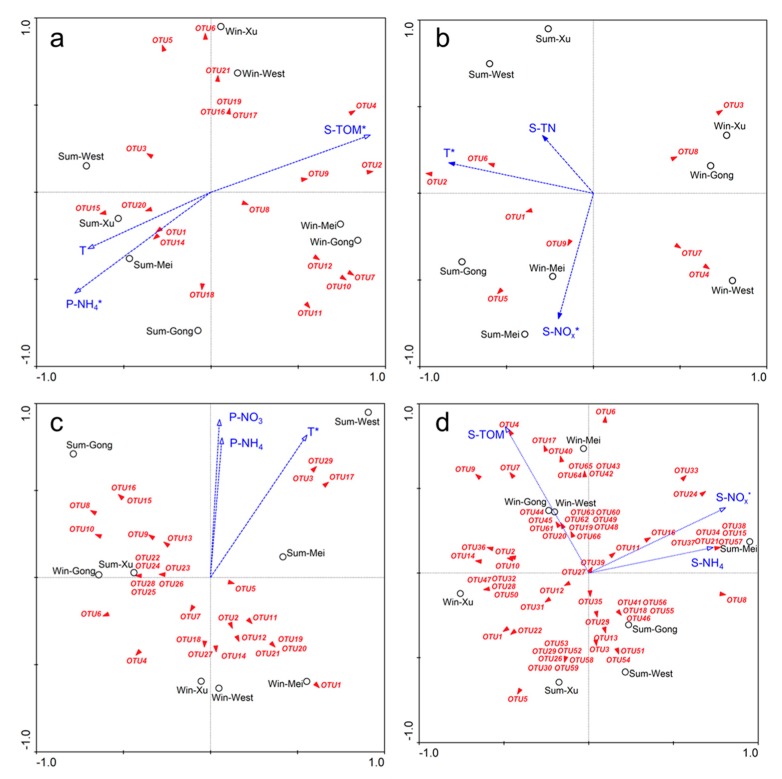
Canonical correspondence analysis (CCA) ordination plots for the relationship between ammonia-oxidizing bacteria (**a**), ammonia-oxidizing archaea (**b**), anammox bacteria (**c**), denitrifying bacteria (**d**) community composition with the environmental parameters in the different lake regions of Lake Taihu.

According to the CCA analysis for the AOB, S-TOM (*p* < 0.05) and P-NH_4_ (*p* < 0.05) were significantly correlated with the variation in AOB community, and these two environmental parameters on these two axes explained 60.3% of the AOB community variance (Figure 8a). For the AOA, S-NO_x_ (*p* < 0.05) and T (*p* < 0.05) were significantly correlated with the variation in AOA community, and these 2 environmental parameters on these two axes explained 68.5% of the AOA community variance (Figure 8b). For the AAOB, T (*p* < 0.05) was significantly correlated with the variation in AAOB community, and the environmental parameter on the two axes explained 46.3% of the AAOB community variance (Figure 8c). For the DNB, S-NO_x_ (*p* < 0.05) was significantly correlated with the variation in DNB community, and the environmental parameter on the two axes explained 39.2% of the DNB community variance (Figure 8d). Besides, the relationship between the AOB community and T, the AOA community and S-TN, the AAOB community and P-NH_4_, P-NO_3_, the DNB community and S-TOM, S-NH_4_ were weak (*p* < 0.1). 

## 4. Discussion

### 4.1. Distribution Characteristics of Nitrogen Occurrence Forms

Nitrogen in overlying water, pore water, and sediment was transferred and transformed under different conditions. On the one hand, nitrogen in pore water and sediment was exchanged with overlying water through molecular diffusion, which affected the occurrence form and characteristics of nitrogen in overlying water. Also, sediment had an adsorption effect on nitrogen in pore water and overlying water. Driven by the concentration gradient, the nitrogen in the sediment and pore water diffused to the overlying water [27]. Therefore, the forms and contents of nitrogen in various media could influence each other through migration and transformation.

The variation trend of nitrogen related index in the sediment and water environments was similar, indicating that there was a strong nitrogen exchange between the two compartments. The endogenous nutrients entered the overlying water from the sediments mainly through the resuspension process of surface sediments and the diffusion of pore water. Some scholars consider the process of nitrogen release from sediments into the overlying water a mechanism that allows nutrient supply to the overlying water [28]. Therefore, while the exogenous source was effectively controlled, the endogenous release could not be ignored.

### 4.2. The Relation between Functional Gene Abundance and Nitrogen Occurrence Characteristic

The abundance of nitrogen-transforming genes significantly affected the occurrence characteristic and distribution patterns of nitrogen in sedimentary environment. Except for A4, the abundance of the *nirS* gene in the sediments increased with a decrease in the trophic status, which resulted in a greater NO_3_^-^-N transformation potential and a lower NO_3_^−^-N concentration in the mesotrophic lake region.

In three typical lake bays of Lake Taihu (A1, A2, and A3), the abundance of the *Arch-amoA*, *amoA*, and *hzo* genes decreased with the drop of trophic status, indicating a strong NH_4_^+^-N transformation potential (ammonia oxidation and anammox) in the eutrophic lake region. However, the concentration of NH_4_^+^-N was higher in the eutrophic lake region, which could be a result of the mineralization of organic nitrogen in the sediments. Wu et al. (2015) used an isotope technique to study nitrogen sources and found that NH_4_^+^-N in the sediments of Lake Taihu is primarily derived from the mineralization of organic nitrogen [29]. In the study area, organic nitrogen was the primary form of nitrogen in the sediments (Figure S1-c–e), accounting for nearly 90%, and organic nitrogen in the sediments was transformed via ammoniation by the action of the microorganisms. The main source of organic matter in the mesotrophic lake region (A3) was aquatic plants, which are rich in cellulose and poor in nitrogen, while the main source of organic matter in the eutrophic lake regions (A1, A2, and A4) were algal remains, which are rich in protein and nitrogen. Therefore, the content of NH_4_^+^-N in the sediments of the eutrophic lake regions was higher than in the mesotrophic lake region with microbial activity.

The abundance of the *Arch-amoA* gene was higher than the *hzo* gene in A1, A2, and A3, while the abundance of the *hzo* gene was higher than the *Arch-amoA* gene in A4, which may due to the organic pollution. Several studies have recently indicated that a high concentration of organic pollutants could drive the anammox bacterial utilization efficiency of NO_2_^−^-N [30]. At the same time, organic matter can be an electron donor when the concentration is relatively high, which promotes the release of NH_4_^+^-N through dissimilatory nitrate reduction [31]. Moreover, Wu et al. (2010) reported that the abundance of the *Arch-amoA* gene was negatively related to the concentration of organic matter [17]. Western Lake Taihu was the primary inflow area of Lake Taihu, and the upstream water contains considerable organic pollutant wastewater, which resulted in a high abundance of the *hzo* gene and a relatively low abundance of the *Arch-amoA* gene.

Conclusions regarding the abundance of the *Arch-amoA* and *amoA* genes in different environments were controversial. The abundance of *Arch-amoA* gene was significantly higher than the *amoA* gene in freshwater lakes, such as the Qiantang River and the Zhujiang River [32,33]. In contrast, the abundance of the *amoA* gene was significantly higher than *Arch-amoA* gene in saltwater lakes, such as the tidal flats of Chongming Island and Qinghai Lake [34], which implies that the abundance of the *Arch-amoA* and *amoA* genes was closely related to salinity. In this study, the abundance of the *Arch-amoA* gene was two orders of magnitude higher than that of *amoA* gene in Lake Taihu, which could be due to ammonia oxidation archaeal preference of a low NH_4_^+^-N environment [17], which was confirmed by the same result in an oligotrophic natural wetland [35]. Additionally, the abundance of the *Arch-amoA* gene and the *amoA* gene were almost of the same order of magnitude in the sediments of a eutrophic freshwater aquaculture area in which NH_4_^+^-N concentration was high [36].

### 4.3. Effect of Trophic Status on the Microbial Community Distribution Characteristics

In this study, two AOBs dominated by *Nitrosomonas* were found in the sediments of Lake Taihu, and the sediments of the eutrophic Qiantang River had a similar distribution characteristic to AOB [32]. *Nitrosomonas* were reported as the primary AOB community in many areas, such as the Elbe River estuary [37] and the freshwater area of Schelde Bay [38], which indicated they survived extensively in different environments. The proportion of *Nitrosomonas* in A1, A2, and A4 was higher than in A3, which may be related to the physicochemical characteristics of sediments. A high concentration of NH_4_^+^-N has been reported as a favorable growth environment for *Nitrosomonas* [39], and *Nitrosomonas* was frequently found in NH_4_^+^-N concentrated wastewater [40]. Wei (2011) also reported that *Nitrosomonas* was the primary AOB community in the rhizosphere soil of three aquatic plants in a eutrophic reservoir [41]. In the sediments of A3, the relative proportion of *Nitrosospira,* which was the other predominant AOB community, was higher than for the other three lake regions, probably because *Nitrosospira* have more competitive survival ability than *Nitrosomonas* in an area with a low NH_4_^+^-N concentration [42].

The AOA *Arch-amoA* gene sequence in the sediments of Lake Taihu was highly similar to other lacustrine environments [43]. The predominant AOA in the research area was *Nitrosopumilus maritimus*, which was a chemoautotroph that used NH_4_^+^-N as the only energy source for growth. The uptake efficiency for NH_4_^+^-N of *Nitrosopumilus maritimus* was very high, and the minimum NH_4_^+^-N concentration for ammonia oxidation by *Nitrosopumilus maritimus* was more than 100 times lower than the AOB [39]. As a result, the AOA possess absolute superiority when competing with other microbes for transforming ammonium nitrogen, which explained the considerably higher abundance of the AOA *amoA* gene than in the AOB, even in a low NH_4_^+^-N concentration environment. At the same time, the affinity for NH_4_^+^-N and *Nitrosopumilus maritimus* resulted in a non-significant difference of the AOA community structure in each lake region of Lake Taihu. *Nitrosopumilus maritimus* was only found in winter samples, possibly because of their preference for lower temperatures. Previous studies of the tidal area of Chongming Island also revealed a similar seasonal distribution pattern as this study [34].

The predominant AAOB in Lake Taihu were *Brocadia* and *Anammoxoglobus*, which was the same conclusion arrived at in a previous study of the sediments of the eutrophic DongJiang River [12]. *Brocadia* was the most common and predominant AAOB in the river and lake sediments, such as in the Xinyi River in Jiangsu Province, China [44] and the Qiantang River in Zhejiang Province, China [30]. *Anammoxoglobus* had a high affinity for the substrate in the sediments, which can confer a great competitive advantage with other AAOB when organic matter and NH_4_^+^-N are simultaneously present [45]. A small amount of *Kuenenia* and *Scalindua* were found in the A2 sediments, which primarily occurs in marine and estuarine eco-systems [46]. *Scalindua* has a relatively high tolerance for salinity, therefore, its distribution was highly driven by salinity. On the other hand, *Kuenenia* was detected in the soil, in a chemical polluted estuary and in activated sludge [7,30], which may explain the very low amount of *Kuenenia* and *Scalindua* in the freshwater Lake Taihu eco-system. Another common AAOB (*Jettenia)* was not found in the study area, possibly because of its preference for high water quality in freshwater river eco-systems and groundwater [46]. In addition, this research found that a considerable amount of OTUs in the AAOB phylogenetic tree belonged to Cluster AAOB A-E, and the proportion was greatest in A2 and A4. This finding indicated that the appearance of new AAOB species in those areas was possible under the long-term effect of the water diversion project “Diverting of Yangzte River water to Lake Taihu” and the upstream inflow.

In this study, many sequences in the *nirS* gene clone library had high similarity with those in the high nitrogen estuaries of the Yangzte River and the Zhujiang River, which indicated that the variety of the DNB community structure was possibly related to the nitrogen concentration. Most of the sequences in *nirS* gene clone library can be classified into *Bacteroidetes*, *Actinobacteria*, and *Gammaproteobacteria*, which were consistent with the previous results for DNB taxonomy [47]. *Halomonas* is a saline-tolerant moderately halophilic bacterium that lives in extreme environments and is primarily distributed in waters with high salinity, pollution and high alkalinity [48]. Berendes et al. (1996) isolated a denitrifying bacterium belonging to *Halomonas* in a wastewater treatment plant and observed that this strain had strong denitrification ability [49]. *Flavobacterium* is a facultatively anaerobic bacteria that can use NO_3_^−^-N and NO_2_^−^-N as an electron acceptor for anaerobic respiration under anoxic and anaerobic conditions and also can degrade organic pollutants [50]. Certain species of *Pseudomonas* and *Arthrobacter* can denitrify under oxic conditions. Qiang et al. (2010) isolated a strain of oxic denitrifying bacteria from river sediments and identified it as *Pseudomonas* sp., which had a high degradation efficiency for TN and NO_3_^-^-N [51]. He et al. (2016) discovered that *Arthrobacter* can utilize a single nitrogen source for oxic denitrification, with a removal efficiency of NO_3_^−^-N exceeding 60% [52].

### 4.4. Factors Affecting Nitrogen-Transforming Microbial Community Structure

The NH_4_^+^-N in the pore water was the main factor that affected the AOB community structure. At the same time, the diversity of the AOB community in the eutrophic lake region was universally higher than that in the mesotrophic lake region, which was primarily due to the differences of the NH_4_^+^-N and TN concentrations and has been confirmed by other researchers [34]. Temperature is another important factor that affects the AOB community structure. In this study, the seasonal variation of the AOB community structure was significant, and the diversity in the winter was higher than in the summer, which was consistent with previous results for wetlands of the Hongkong Mipu Nature Reserve area [8]. Several studies have also indicated that the relative abundance of some AOB was higher at low temperatures (4–10 °C) and lower at high temperatures (>30 °C) [53]. The AOB was typical inorganic chemoautotrophic bacteria such that the organic matter in the soil was sufficient to provide the preferred pH and aeration conditions, which would promote the growth of the AOB. This conclusion has also been confirmed by a study of the AOB abundance in three different soils, which determined that the relative abundance of the AOB was higher in soil with sufficient organic matter [54].

The temperature was also an important factor that affected the AOA community structure. A study of estuary sediments in San Francisco Bay indicated that temperature was significantly related to the AOA community structure [9]. It has also been reported that the AOA was much more tolerant to a wide temperature range than the AOB [55]. From the viewpoint of species revolution, AOA can better survive and revolute in an extreme environment (i.e., arctic and extremely hot), similar to on the early earth, and play a leading role in the ammonia oxidation process. As the substrate of the ammonia oxidation process, the NO_x_^−^-N concentration could also influence the AOA community structure. A study of the AOA community structure in the sediments of a Florida estuary indicated that the NO_2_^−^-N concentration was positively related to the amount of *Thaumarchaeota* [10].

It has been reported that NH_4_^+^-N is a substrate for anaerobic ammonia oxidation and was the governing factor for the AAOB community structure in a Qiantang River sediments [30]. Although NO_3_^−^-N could not be utilized by the AAOB, it has been reported as an important environmental factor that affects the AAOB community structure in sediments of the Mai Po Nature estuary and Jiaozhou Bay [11,30]. In other environments, such as the southern China Sea and a wastewater treatment plant, the abundance of the AAOB had a positive relationship with the NO_3_^−^-N concentration [56].

NO_3_^−^-N is an important factor that affected the DNB community. In a soil cultivation experiment, where NO_3_^−^-N was added, the denitrification rate increased rapidly during the initial stage and the *nirK*-type DNB community structure did not change considerably, but its relative abundance obviously increased, and the *nirS*-type DNB were not affected. After 20 days, the community structure and the abundance of the *nirK*-type DNB did not respond to NO_3_^−^-N, while the *nirS*-type DNB community structure was considerably changed, and its relative abundance also increased with an increase in the NO_3_^−^-N concentration [57]. Organic matter could also affect the DNB community structure, as indicated by a report that acetic acid could be a selective factor for DNB, and ethanol or methanol could induce the appearance of a unique DNB community [58].

## 5. Conclusions

Nitrogen-transforming genes (*amoA*, *Arch-amoA*, *nirS*, and *hzo*) were used to investigate the bacterial community and gene abundance in Lake Taihu. A total of 768 clones were analyzed in the context of changing environmental conditions to evaluate the impact of trophic status on bacterial community. The findings of the current study revealed that (1) Except for region A4, *nirS* gene abundance was inversely related to the trophic levels of the water body seasons of the year, and the abundance of *amoA*, *Arch-amoA* and *hzo* genes changed in the opposite direction. (2) *Nitrosomonas* was the predominant AOB in Lake Taihu and was more significantly predominant in the eutrophic lake regions. The predominant AOA and DNB were *Nitrosopumilus maritimus* and *Flavobacterium*, respectively. *Brocadia* and *Anammoxoglobus* were the two predominant AAOBs in Lake Taihu. (3) The major environmental factors affecting nitrogen-transforming bacterial community compositions were determined to be NH_4_^+^-N in pore water as well as TOM and NO_x_^-^-N in sediments.

## Figures and Tables

**Table 1:** Diversity properties of the nitrogen-transforming genes clone libraries from different lake regions.

Genes	Lake Region	Summer				Winter			
		A1	A2	A3	A4	A1	A2	A3	A4
*nirS*	The number of clones	26	24	28	25	29	29	29	28
	The number of OTUs	12	14	14	12	17	18	14	19
	Shannon	3.32	3.55	3.54	3.15	3.82	3.92	3.66	4.18
	Coverage (%)	80.8	62.5	75.0	72.0	62.1	58.6	86.2	53.6
*amoA*	The number of clones	13	24	14	14	30	30	31	29
	The number of OTUs	8	9	4	5	10	8	7	11
	Shannon	2.82	2.43	1.57	2.07	2.98	2.55	2.66	2.99
	Coverage (%)	61.5	79.2	85.7	85.7	86.7	93.3	96.8	75.9
*Arch-amoA*	The number of clones	28	33	30	28	21	21	20	20
	The number of OTUs	5	3	4	4	5	3	4	4
	Shannon	1.91	1.35	1.59	1.47	1.88	1.03	1.68	1.80
	Coverage (%)	96.4	97.0	96.7	96.4	95.2	95.2	95.0	95.0
*hzo*	The number of clones	23	18	18	22	20	18	20	25
	The number of OTUs	9	10	8	5	8	9	10	7
	Shannon	2.94	3.24	2.71	1.43	2.62	2.86	2.95	2.45
	Coverage (%)	87.0	83.3	77.8	86.4	90.0	72.2	70.0	96.0

Operational taxonomic units ( OTUs) were defined at 3% nucleotide acid divergence.

## Supplementary Materials

The following are available online at https://www.mdpi.com/1660-4601/16/13/2298/s1, Figure S1: Physiochemical properties of the pore water and sediment in different trophic statuses lake regions in four seasons., Table S1: physicochemical parameters of the overlying water, pore water, and freeze dried sediments, Table S2: PCR primers used in the present study, Table S3: The trophic status index and trophic state evaluation in the research area, Table S4: The abundance of nirS gene in each sampling site (with 3 duplicates) during 4 seasons, Table S5: The abundance of amoA gene in each sampling site (with 3 duplicates) during 4 seasons, Table S6: The abundance of Arch-amoA gene in each sampling site (with 3 duplicates) during 4 seasons, Table S7: The abundance of hzo gene in each sampling site (with 3 duplicates) during 4 seasons.
