# Supplementary material for: Spatial and Seasonal Variations in the Abundance of Nitrogen-Transforming Genes and the Microbial Community Structure in Freshwater Lakes with Different Trophic Statuses
Source: Int J Environ Res Public Health. 2019 Jun 28;16(13):2298. doi: 10.3390/ijerph16132298 (PMC6651097; doi:10.3390/ijerph16132298)
Supplement: Supplementary file 1 [file ijerph-16-02298-s001.pdf]

Supplementary Information

Table S1. physicochemical parameters of the overlying water, pore water, and freeze dried sediments.

	Physicochemical Parameters	Determination Method
Sediment	Total nitrogen (S-TN)	Micro-Kjeldahl method
	Ammonium nitrogen ($S\text{-}NH_4$)	Devarda alloy distillation method
	Nitrate-nitrite nitrogen ($S\text{-}NO_x$)	Devarda alloy distillation method
	Total phosphorus (S-TP)	Molybdenum-blue colorimetry
	Total organic matter (S-TOM)	Dichromate oxidation method
Overlying water	Total nitrogen (W-TN)	Alkaline potassium persulfate digestion UV Spectrophotometric method
	Total phosphorus (W-TP)	Ammonium molybdate spectrophotometric method
	Chemical Oxygen Demand (COD)	Dichromate method
	Temperature (T)	Multi-parameter water quality sonde (YSI, 6600-V2, Yellow Springs, OH, USA)
	Chlorophyll a (Chla)	
	pH	
	Secchi Disc (SD)	
Porewater	Ammonium ($P\text{-}NH_4$)	Nessler's reagent spectrophotometry
	Nitrate nitrogen ($P\text{-}NO_3$)	Ultraviolet spectrophotometry

Table S2. PCR primers used in the present study

Primer	5′ - 3′	References
hzoF1	TGTGCATGGTCAATTGAAAG	Li et al., 2010
hzoR1	CAACCTCTTCWGCAGGTGCATG	
amoA1f	GGGGTTTCTACTGGTGGT	Rotthauwe et al., 1997
amoA2r	CCCCTCKGSAAAGCCTTCTTC	
Arch-amoAF	STAATGGTCTGGCTTAGACG	Francis et al., 2005
Arch-amoAR	GCGGCCATCCATCTGTATGT	
nirS3F	CCTA(C/T)TGGCCGCC(A/G)CA(A/G)T	Braker et al., 1998
nirS5R	GCCGCCGTC(A/G)TG(A/C/G)AGGAA	

Table S3. The trophic status index and trophic state evaluation in the research area

Lake	Trophic Status Index (*TSI*)				Trophic State
	Spring	Summer	Autumn	Winter	
Meiliang Bay	60.8	61.9	60.8	60.6	medium eutrophication
Gonghu Bay	56.5	56.7	58.3	53.6	light eutrophication
Xukou Bay	48.5	49.0	42.2	41.3	mesotrophication
Western Lake Taihu	62.4	62.7	61.9	58.3	Medium/ light eutrophication

Table S4. The abundance of *nirS* gene in each sampling site (with 3 duplicates) during 4 seasons

	Spring	Summer	Autumn	Winter
A1-1	2.88×10^8	4.61×10^8	3.38×10^8	3.81×10^8
	3.07×10^8	5.60×10^8	4.13×10^8	5.27×10^8
	3.64×10^8	6.26×10^8	5.01×10^8	5.56×10^8
A1-2	2.55×10^8	5.64×10^8	5.07×10^8	3.51×10^8
	2.72×10^8	6.84×10^8	6.20×10^8	4.86×10^8
	3.23×10^8	7.65×10^8	7.51×10^8	5.13×10^8
A2-1	4.44×10^8	6.57×10^8	5.17×10^8	4.73×10^8
	4.74×10^8	7.98×10^8	6.32×10^8	6.54×10^8
	5.63×10^8	8.92×10^8	7.66×10^8	6.91×10^8
A2-2	4.10×10^8	6.31×10^8	4.97×10^8	4.19×10^8
	4.37×10^8	7.67×10^8	6.07×10^8	5.80×10^8
	5.19×10^8	8.57×10^8	7.36×10^8	6.12×10^8
A3-1	4.74×10^8	1.30×10^9	7.74×10^8	7.57×10^8
	5.76×10^8	1.58×10^9	9.46×10^8	1.05×10^9
	6.44×10^8	1.76×10^9	1.15×10^9	1.11×10^9
A3-2	5.35×10^8	1.20×10^9	6.86×10^8	5.26×10^8
	6.49×10^8	1.46×10^9	8.39×10^8	7.28×10^8
	7.26×10^8	1.63×10^9	1.02×10^9	7.69×10^8
A4-1	2.16×10^8	3.79×10^8	2.04×10^8	2.23×10^8
	2.62×10^8	4.60×10^8	2.50×10^8	3.09×10^8
	2.93×10^8	5.14×10^8	3.03×10^8	3.26×10^8
A4-2	2.44×10^8	3.36×10^8	3.06×10^8	1.49×10^8
	2.96×10^8	4.08×10^8	3.75×10^8	2.06×10^8
	3.31×10^8	4.56×10^8	4.54×10^8	2.17×10^8

Table S5. The abundance of *amoA* gene in each sampling site (with 3 duplicates) during 4 seasons

	Spring	Summer	Autumn	Winter
A1-1	2.97×10^6	2.04×10^6	5.96×10^5	5.94×10^6
	3.17×10^6	2.48×10^6	7.28×10^5	8.22×10^6
	3.76×10^6	2.77×10^6	8.83×10^5	8.68×10^6
A1-2	2.63×10^6	1.67×10^6	8.94×10^5	6.43×10^6
	2.81×10^6	2.03×10^6	1.09×10^6	8.91×10^6
	3.34×10^6	2.27×10^6	1.32×10^6	9.40×10^6
A2-1	1.90×10^6	7.27×10^5	9.55×10^5	3.36×10^6
	2.03×10^6	8.82×10^5	1.17×10^6	4.65×10^6
	2.41×10^6	9.86×10^5	1.41×10^6	4.91×10^6
A2-2	1.75×10^6	7.56×10^5	9.17×10^5	2.98×10^6
	1.87×10^6	9.18×10^5	1.12×10^6	4.13×10^6
	2.22×10^6	1.03×10^6	1.36×10^6	4.36×10^6
A3-1	4.63×10^5	2.91×10^5	6.18×10^5	1.78×10^6
	5.63×10^5	3.53×10^5	7.55×10^5	2.46×10^6
	6.29×10^5	3.95×10^5	9.15×10^5	2.60×10^6
A3-2	5.23×10^5	2.69×10^5	5.48×10^5	2.56×10^6
	6.35×10^5	3.26×10^5	6.69×10^5	3.54×10^6
	7.09×10^5	3.64×10^5	8.11×10^5	3.74×10^6
A4-1	6.90×10^5	1.31×10^5	3.46×10^5	7.98×10^5
	8.38×10^5	1.59×10^5	4.23×10^5	1.10×10^6
	9.37×10^5	1.78×10^5	5.13×10^5	1.17×10^6
A4-2	6.12×10^5	1.48×10^5	2.31×10^5	5.32×10^5
	7.43×10^5	1.80×10^5	2.82×10^5	7.36×10^5
	8.31×10^5	2.01×10^5	3.42×10^5	7.77×10^5

Table S6. The abundance of *Arch-amoA* gene in each sampling site (with 3 duplicates) during 4 seasons

	Spring	Summer	Autumn	Winter
A1-1	4.42×10^8	1.15×10^8	2.46×10^8	2.25×10^8
	4.72×10^8	1.40×10^8	3.01×10^8	3.12×10^8
	5.60×10^8	1.56×10^8	3.65×10^8	3.29×10^8
A1-2	4.99×10^8	9.42×10^7	1.64×10^8	2.44×10^8
	5.32×10^8	1.14×10^8	2.01×10^8	3.38×10^8
	6.32×10^8	1.28×10^8	2.43×10^8	3.57×10^8
A2-1	2.70×10^8	6.57×10^7	4.62×10^7	1.44×10^8
	2.88×10^8	7.97×10^7	5.64×10^7	1.99×10^8
	3.42×10^8	8.91×10^7	6.84×10^7	2.10×10^8
A2-2	2.49×10^8	6.83×10^7	4.43×10^7	1.62×10^8
	2.65×10^8	8.30×10^7	5.42×10^7	2.25×10^8
	3.15×10^8	9.28×10^7	6.57×10^7	2.37×10^8
A3-1	3.46×10^7	2.08×10^7	7.47×10^6	7.01×10^7
	4.20×10^7	2.53×10^7	9.13×10^6	9.71×10^7
	4.69×10^7	2.83×10^7	1.11×10^7	1.02×10^8
A3-2	3.90×10^7	1.92×10^7	6.62×10^6	4.87×10^7
	4.74×10^7	2.34×10^7	8.09×10^6	6.75×10^7
	5.29×10^7	2.61×10^7	9.81×10^6	7.12×10^7
A4-1	7.22×10^6	5.92×10^6	1.43×10^6	1.95×10^7
	8.77×10^6	7.19×10^6	1.75×10^6	2.69×10^7
	9.80×10^6	8.04×10^6	2.12×10^6	2.84×10^7
A4-2	6.41×10^6	5.25×10^6	9.52×10^5	1.30×10^7
	7.78×10^6	6.38×10^6	1.16×10^6	1.80×10^7
	8.69×10^6	7.13×10^6	1.41×10^6	1.90×10^7

Table S7. The abundance of *hzo* gene in each sampling site (with 3 duplicates) during 4 seasons

	Spring	Summer	Autumn	Winter
A1-1	5.12×10^6	4.13×10^7	6.68×10^6	6.26×10^6
	5.46×10^6	5.02×10^7	8.16×10^6	8.66×10^6
	6.48×10^6	5.61×10^7	9.90×10^6	9.15×10^6
A1-2	4.54×10^6	3.38×10^7	1.00×10^7	6.78×10^6
	4.84×10^6	4.10×10^7	1.22×10^7	9.39×10^6
	5.75×10^6	4.59×10^7	1.48×10^7	9.91×10^6
A2-1	8.48×10^5	2.17×10^7	5.98×10^6	4.64×10^6
	9.04×10^5	2.63×10^7	7.30×10^6	6.43×10^6
	1.07×10^6	2.94×10^7	8.85×10^6	6.78×10^6
A2-2	9.18×10^5	2.26×10^7	6.22×10^6	4.12×10^6
	9.80×10^5	2.74×10^7	7.60×10^6	5.70×10^6
	1.16×10^6	3.06×10^7	9.22×10^6	6.02×10^6
A3-1	2.09×10^5	2.94×10^6	7.67×10^5	5.69×10^5
	2.54×10^5	3.57×10^6	9.37×10^5	7.88×10^5
	2.84×10^5	3.99×10^6	1.14×10^6	8.32×10^5
A3-2	1.86×10^5	3.19×10^6	6.80×10^5	3.96×10^5
	2.26×10^5	3.87×10^6	8.31×10^5	5.48×10^5
	2.52×10^5	4.33×10^6	1.01×10^6	5.78×10^5
A4-1	2.62×10^7	7.24×10^7	4.83×10^7	2.61×10^7
	3.18×10^7	8.80×10^7	5.90×10^7	3.62×10^7
	3.55×10^7	9.83×10^7	7.16×10^7	3.82×10^7
A4-2	2.95×10^7	6.42×10^7	3.22×10^7	1.74×10^7
	3.58×10^7	7.80×10^7	3.94×10^7	2.41×10^7
	4.00×10^7	8.72×10^7	4.77×10^7	2.55×10^7

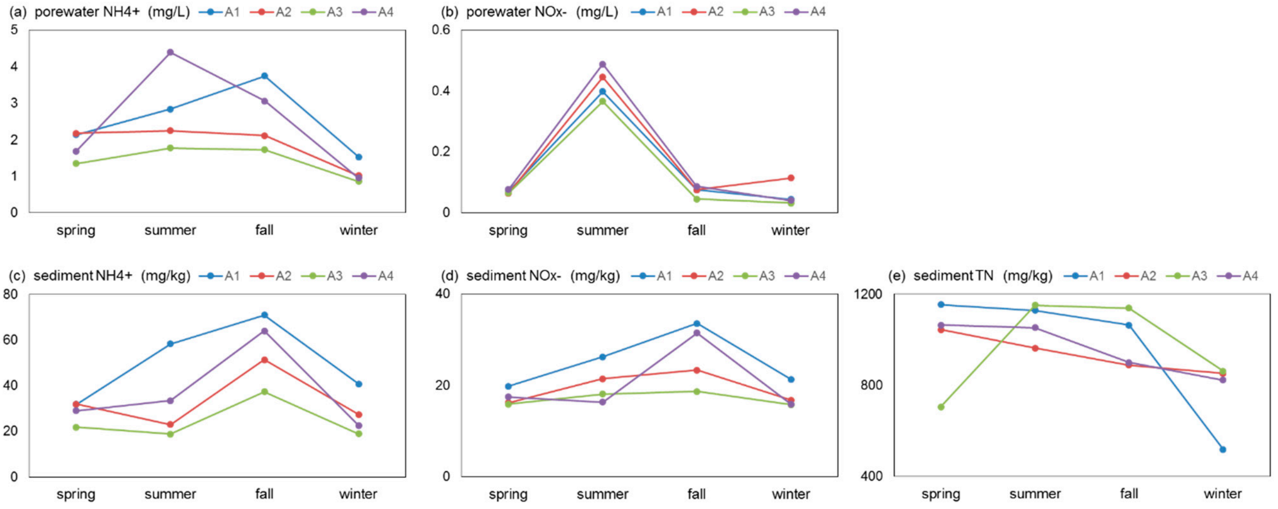

Figure S1. Physiochemical properties of the pore water and sediment in different trophic statuses lake regions in four seasons. (**a**), NH_4^+-N in pore water; (**b**), NO_x^--N in pore water; (**c**), NH_4^+-N in sediment; (**d**), NO_x^--N in sediment; (**e**), TN in sediment.

References

1 Li, H.; Chen, S.; Mu, B.Z.; Gu, J.D. Molecular detection of anaerobic ammonium-oxidizing (anammox) bacteria in high-temperature petroleum reservoirs. *Microb. Ecol.* **2010**, *60*, 771–783, doi:10.1007/s00248-010-9733-3.

2 Rotthauwe, J.H.; Witzel, K.P.; Liesack, W. The ammonia monooxygenase structural gene *amoA* as a functional marker: Molecular fine-scale analysis of natural ammonia-oxidizing populations. *Appl. Environ. Microbiol.* **1997**, *63*, 4704–4712. Available online: https://aem.asm.org/content/63/12/4704.short (accessed on 27 June 2017).

3 Francis, C.A.; Roberts, K.J.; Beman, J.M.; Santoro, A.E.; Oakley, B.B. Ubiquity and diversity of ammonia-oxidizing archaea in water columns and sediments of the ocean. *Proc. Natl. Acad. Sci. USA* **2005**, *102*, 14683–14688, doi:10.1073/pnas.0506625102.

4 Braker, G.; Fesefeldt, A.; Witzel, K.P. Development of PCR primer systems for amplification of nitrite reductase genes (nirK and nirS) to detect denitrifying bacteria in environmental samples. *Appl. Environ. Microbiol.* **1998**, *64*, 3769–3775. Available online: https://aem.asm.org/content/64/10/3769.short (accessed on 27 June 2017).
